# The Impact of Lesion Complexity and the CHA_2_DS_2_-VASc Score on Spontaneous Reperfusion in Patients with ST-Segment Elevation Myocardial Infarction

**DOI:** 10.1155/2022/8066780

**Published:** 2022-02-09

**Authors:** Gökhan Alıcı, Hasan Ali Barman, Adem Atıcı, Sevil Tuğrul, Ömer Genç, İrfan Şahin

**Affiliations:** ^1^Okmeydani Training and Research Hospital, Department of Cardiology, Darulaceze Street No:25, Okmeydanı 34384, İstanbul, Turkey; ^2^İstanbul University–Cerrahpasa, Institute of Cardiology, İstanbul, Turkey; ^3^İstanbul Medeniyet University, Göztepe Training and Research Hospital, Department of Cardiology, İstanbul 34722, Turkey; ^4^Bağcılar Training and Research Hospital, Department of Cardiology, Bağcılar Center, Mimar Sinan Street, Bağcılar, İstanbul 34100, Turkey; ^5^Ağrı Training and Research Hospital, Department of Cardiology, Ağrı Center, Ağrı 04200, Turkey

## Abstract

**Background:**

In patients with ST-segment elevation myocardial infarction (STEMI) undergoing primary percutaneous coronary intervention (PCI), a patent infarct-related artery (IRA) on initial angiography is defined as spontaneous reperfusion (SR).

**Objective:**

The present study aimed to determine the impact of lesion complexity and the CHA_2_DS_2_-VASc score on SR in patients with STEMI.

**Methods:**

A total number of 1,641 consecutive patients with STEMI undergoing primary PCI were assessed for this study. Patients were divided into 2 groups, those with SR, SR(+) (*n* = 239), and those without SR, SR(−) (*n* = 1402), according to their initial angiography and SR status. CHA_2_DS_2_-VASc scores were calculated for all patients. The lesion complexity of coronary artery disease was assessed with the SYNTAX score.

**Results:**

The CHA_2_DS_2_-VASc and SYNTAX scores were significantly lower in the SR(+) group compared to the SR(−) (mean CHA_2_DS_2_-VASc, 1.36 ± 0.64 vs. 2.01 ± 0.80, *p* < 0.001; mean SYNTAX score, 15.51 ± 5.94 vs. 17.08 ± 8.29, *p* < 0.001). After the multivariate regression analysis, a lower CHA_2_DS_2_-VASc (OR = 0.288, *p* < 0.001), SYNTAX score (OR = 0.920, *p*=0.007), uric acid (OR = 0.868, *p*=0.005), CRP (OR = 0.939, *p*=0.001), BNP (OR = 0.998, *p*=0.004), and troponin (OR = 0.991, *p*=0.001) were independent predictors of SR. In-hospital mortality rates were significantly lower in the SR(+) group compared to the SR(−) (0% vs. 6.7%, *p* < 0.001).

**Conclusion:**

Our study demonstrated that lesion complexity and the CHA_2_DS_2_-VASc score are independently associated with spontaneous reperfusion.

## 1. Introduction

Acute coronary syndromes (ACS) are the leading causes of death worldwide [1]. The plaque rupture and erosion followed by thrombus formation within the vessel lumen are significant etiologies in the pathophysiology of ACS [2]. The absence of collateral perfusion in the setting of complete coronary occlusion results in acute ST-segment elevation myocardial infarction (STEMI). STEMI patients initially undergo cardiac catheterization after being admitted to the hospital and are often evaluated and treated with percutaneous coronary intervention (PCI). PCI is a first-choice reperfusion therapy for STEMI patients if an experienced team is available to intervene promptly. Guidelines recommend a maximum expected delay of 60 min from STEMI diagnosis to primary PCI (wire crossing) at hospitals capable of primary PCI [3, 4]. Besides, thrombolytic therapy has been an alternative reperfusion strategy if primary PCI cannot be performed timely. In such patients, the primary goal of treatment is to prevent myocardial necrosis after achieving reperfusion. Meaningfully, reperfusion is of prime importance for an ischemic tissue to maintain its vitality. Spontaneous reperfusion (SR) is defined as the achievement of initial thrombolysis in myocardial infarction (TIMI) grade 3 flow in the infarction-related artery (IRA) before reperfusion therapy. SR is seen in 7% to 27% of patients with acute STEMI [5]. In STEMI patients, the achievement of early TIMI grade 3 flow in the IRA increases the ventricular performance and reduces mortality rate [6]. Among STEMI patients undergoing primary PCI, those with SR on coronary angiography (CAG) have a better prognosis than patients without spontaneous blood flow [7, 8].

Despite many related risk classification systems described in the literature, there is no standardized approach to the prediction of SR. The CHA_2_DS_2_-VASc score is a simple, easy-to-remember, valid, and practical scoring system that is used for estimating cardioembolic risk in patients with nonvalvular atrial fibrillation (AF) and for determining indications for anticoagulant therapy [9]. The parameters used in the CHA_2_DS_2_-VASc score also involve the mutual risk factors for coronary artery disease (CAD). Recently, the CHA_2_DS_2_-VASc score has become a predictor of ischemic stroke and cardiovascular events in cases of CAD without AF [10, 11]. Nevertheless, to our knowledge, the relationship between the CHA_2_DS_2_-VASc score and SR is yet to be investigated.

The present study was designed to examine the relationship between the CHA_2_DS_2_-VASc score, lesion complexity and SR in STEMI patients undergoing primary PCI.

## 2. Materials and Methods

The retrospective, cross-sectional study included a total of 1,641 consecutive patients who were admitted to our hospital with a prediagnosis of STEMI within 12 hours from symptom onset and underwent primary PCI, between 2012 and 2017. STEMI was diagnosed in the presence of the following criteria based on the European Society of Cardiology (ESC) Guidelines [12]: (i) new ST-segment elevation at the *J* point in two contiguous leads with the cut-off value greater than 0.1 mV in all leads other than V2 or V3 and (ii) in leads V2-V3, a cut-off value of ≥0.2 mV in men aged ≥40 years, ≥0.25 in men aged <40 years, or ≥0.15 mV in women. SR was defined as the presence of TIMI grade 3 flow in the IRA on baseline CAG.

**Figure 1:**
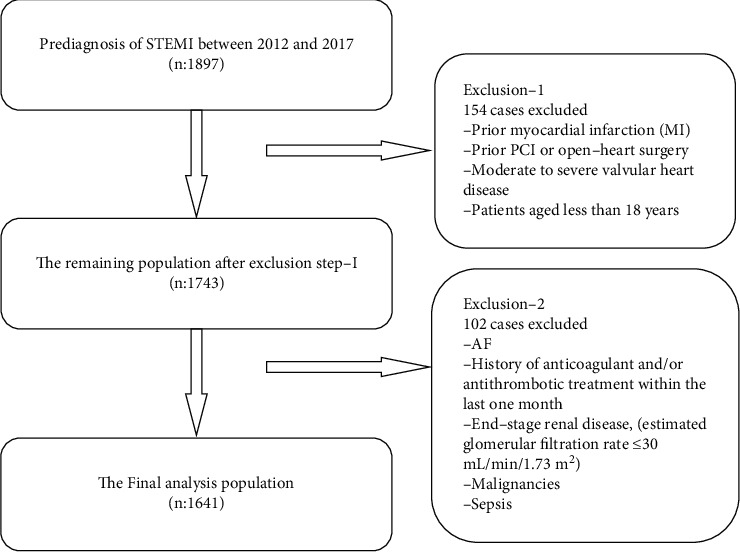
Flowchart of inclusion in the study.

Patients with AF, history of anticoagulant and/or antithrombotic treatment within the last one month, end-stage renal disease (estimated glomerular filtration rate ≤ 30 mL/min/1.73 m^2^), malignancy, sepsis, prior myocardial infarction, prior PCI or open-heart surgery, moderate-to-severe valvular heart disease, and those aged less than 18 years were excluded from the study (Figure 1).

Demographic and clinical characteristics, including age, gender, smoking status, hypertension (HT), diabetes mellitus (DM), hyperlipidemia (HLD), current drug use, and physical examination findings, were recorded for each patient. The laboratory analysis of venous blood samples from all patients at the time of admission to the emergency department was performed using an automated chemistry analyzer (Roche Diagnostic Modular Systems, Tokyo, Japan).

Echocardiographic measurements were performed in accordance with the American Society of Echocardiography (ASE) guidelines using a Vivid 7 GE Medical System (GE Healthcare, Horten, Norway), and the left ventricular ejection fraction (LVEF) was calculated based on the modified Simpson method [13].

Heart failure with reduced EF (HFrEF) was diagnosed in the presence of risk factors, abnormal electrocardiogram, clinical signs (e.g., elevated jugular venous pressure, hepatojugular reflux, and laterally displaced apical impulse), symptoms of heart failure (e.g., reduced exercise tolerance, paroxysmal nocturnal dyspnea, breathlessness, and orthopnoea), and/or objective evidence of reduced LVEF, along with a reduced EF of <50% assessed by echocardiography [14]. Hypertension (HT) was defined as a persistent elevation of systolic blood pressure (SBP) of ≥140 mmHg and/or diastolic blood pressure (DBP) of ≥90 mmHg or a history of antihypertensive drug use. DM was defined as a fasting blood glucose level of ≥126 mg/dL or a history of antidiabetic drug use. Peripheral artery disease (PAD) was defined as the presence of stenosis of >50% in arteries other than the coronary arteries. Vascular disease was defined as the presence of previous myocardial infarction, peripheral artery disease, or aortic plaque.

The CHA_2_DS_2_-VASc and the Synergy Between Percutaneous Coronary Intervention with Taxus and Cardiac Surgery (SYNTAX) I scores were calculated for each patient using the following parameters: HFrEF (C), HT (H), aged over 75 years (A2), DM (D), stroke (S2), vascular disease (V), aged 65 years to 74 years (A), and female gender (sex category [Sc]). The CHA_2_DS_2_-VASc score was calculated by assigning 1 point for each of the parameters including HFrEF, HT, DM, age 65 years to 74 years, female gender, and presence of vascular diseases and by assigning 2 points for the history of stroke and age >75 years [9]. Accordingly, the maximum CHA_2_DS_2_-VASc was calculated as 9 points.

### 2.1. Ethical Statement

All participants gave written informed consent to the study. The study protocol was approved by our institutional ethical committee and was conducted by following the Declaration of Helsinki and good clinical practice guidelines.

### 2.2. Coronary Angiography

All the patients underwent CAG (Siemens Axiom Artis zee 2011; Siemens Health-care, Erlangen, Germany) according to recent guidelines [15]. Angiograms were obtained for the left coronary system in standard 4 different angles (left anterior oblique cranial, left anterior oblique caudal, anteroposterior cranial, right anterior oblique cranial, and right anterior oblique caudal views) and for the right coronary system in standard 2 different angles (left anterior oblique and right anterior oblique) and were recorded to digital memory. The revascularization of IRA for all participants was completed in the same session with CAG. Coronary lesions were evaluated by two experienced interventional cardiologists blinded to all clinical data. A significant and severe coronary artery stenosis was defined as the presence of a lumen reduction of ≥50% in at least one major epicardial coronary artery [16]. The SYNTAX score was calculated before the wiring or predilatation of IRA from the angiographic analysis of coronary lesions with ≥50% stenosis in vessels ≥1.5 mm in diameter, using the original SYNTAX score website (http://www.syntaxscore.com) [17]. The SYNTAX score is calculated by a computer program consisting of 12 sequential and interactive questions that are divided into two groups: the first three questions determine the dominance, the total number of lesions, and the vessel segments involved per lesion. When determining the severity of CAD, the SYNTAX score was calculated based on the total number, functional significance, and localizations of lesions detected on CAG and based on the presence of total occlusion, bifurcation, trifurcation, distal vessel bed, and thrombus formation. In patients with severe CAD, TIMI was graded as follows: grade 0: no perfusion, grade 1: penetration without perfusion, grade 2: partial perfusion, and grade 3: complete perfusion. SR was defined as the achievement of TIMI grade 3 flow in IRA on the baseline CAG [18]. The study population was divided into two groups, the SR (+) and SR (-) groups, based on CAG. The two groups were compared in terms of CHA_2_DS_2_-VASc, demographics, clinical, echocardiographic, and laboratory results.

### 2.3. Intervention Procedure

PCI was chosen as reperfusion therapy for subjects included in the present study. CAG was performed using the standard Judkins technique through the femoral route with a 6–7 Fr guiding catheter. Mostly, drug-coated metal stents were used. All the patients received acetylsalicylic acid (loading dose of 300 mg, maintenance dose of 100 mg), heparin (100 U/kg) intravenously, and a loading dose of 600 mg, followed by a maintenance dose of 75 mg of clopidogrel once every day or a loading dose of 180 mg, followed by a maintenance dose of 90 mg of ticagrelor twice every day or a loading dose of 60 mg, followed by a maintenance dose of 10 mg of prasugrel once every day. Procedural decisions regarding thrombus aspiration and balloon catheter use, stent diameter, length, and type were left to the attending operator's discretion.

### 2.4. Statistical Analysis

All statistical analyses were conducted using the Statistical Package for the Social Sciences 19.0 for Windows (IBM Corp. Released 2010. IBM SPSS Statistics for Windows, Version 19.0. Armonk, NY: IBM Corp.). The normal distribution of data was analyzed using the Kolmogorov–Smirnov test. Continuous data were expressed as mean ± standard deviation (SD) and median (interquartile range; IQR_25–75_). Categorical data were expressed as a number (*n*) and percentage (%). Differences between categorical variables were determined using Fisher's exact test and/or Chi-square test, and unpaired samples were compared using Student's *t*-test or Mann–Whitney *U* test where appropriate. Univariate and multivariate logistic regression analyses were used to identify the independent variables of SR. After performing univariate analysis, significant variables with a *p*-value <0.05 were selected into the multivariate logistic regression analysis using the stepwise method. The results of univariate and multivariate regression analyses were presented as odds ratio (OR) with 95% confidence interval (CI). The optimal cut-off value of the CHA_2_DS_2_-VASc score, which has the highest overall sensitivity and specificity in predicting SR, was determined using the receiver operating characteristic (ROC) curve with the Youden index. The area under the curve (AUC) comparisons of the predictors for SR was performed using the DeLong method [19]. Significance was assumed at a two-sided *p*-value of <0.05.

## 3. Results

Spontaneous reperfusion was detected in 239 out of 1,641 STEMI patients (Table 1). The 239 patients with SR comprised 173 (72.4%) men and 66 (27.6%) women with a mean age of 62.6 ± 12.6 years, and the 1,402 patients without STEMI included 987 (70.4%) men and 415 (29.6%) women with a mean age of 60.5 ± 12.3 years. A significant difference was found between the two groups with regards to age (*p* < 0.05). No significant difference was found between the groups in terms of body mass index (BMI), HT, HLD, and smoking status, whereas the prevalence of DM was significantly higher in the SR group (21 out of 51) compared to the non-SR group (27 out of 391) (*p*=0.035), and the LVEF value was significantly higher in the SR group compared to the non-SR group (41.01 ± 7.51 vs. 36.01 ± 6.63, *p*=0.024). In terms of the antiplatelet treatments received by the patients at the emergency department, a significant difference was found among the patients who received acetylsalicylic acid (ASA) + clopidogrel, ASA + ticagrelor, and ASA + prasugrel, whereas no significant difference was found between the patients who received ASA + ticagrelor and ASA + prasugrel. On the other hand, the CHA_2_DS_2_-VASc and SYNTAX scores were significantly lower in the SR group compared to the non-SR group (mean CHA_2_DS_2_-VASc, 1.36 ± 0.64 vs. 2.01 ± 0.80, *p* < 0.001; mean SYNTAX I score, 15.51 ± 5.94 vs. 17.08 ± 8.29, *p* < 0.001). No significant difference was found between the two groups in the baseline blood parameters, including total cholesterol, low-density lipoprotein (LDL), high-density lipoprotein (HDL), triglyceride, hemoglobin, hematocrit, platelet count, and thyroid-stimulating hormone (TSH) level. In contrast, serum creatinine (Cre) showed a threshold significance level of =0.05, while the uric acid, white blood cell count (WBC), C-reactive protein (CRP), brain natriuretic peptide (BNP), and troponin levels were significantly lower in the SR group compared to the non-SR group (Cre, 0.93 ± 0.35 *vs.* 0.89 ± 0.30, *p*=0.050; uric acid, 4.59 ± 2.05 *vs.* 4.92 ± 1.54, *p*=0.019; WBC, 12.00 ± 3.02 *vs.* 12.87 ± 5.08, *p* < 0.001; CRP, 9.02 ± 4.34 *vs*. 9.95 ± 3.73, *p*=0.002; BNP, 153 (IQR; 50–581) *vs.* 243 (IQR; 60–720), *p*=0.001; troponin, 13 (IQR; 2–50) *vs.* 21 (IQR; 2–50), *p*=0.008) (Table 1).

Parameters that were found to affect SR development in the logistic regression analysis were further analyzed using univariate and multivariate analyses. Univariate analysis was conducted with the parameters including age, gender, BMI, HT, DM, smoking status, antiplatelet therapy received at the emergency department, CHA_2_DS_2_-VASc and SYNTAX scores, uric acid, Cre, TSH, CRP, BNP, and troponin. Of these, age, DM, antiplatelet therapy received at the emergency department, CHA_2_DS_2_-VASc and SYNTAX scores, uric acid, Cre, CRP, BNP, and troponin were found to be statistically significant, and hence, they were further analyzed by multivariate analysis, in which CHA_2_DS_2_-VASc score, SYNTAX score, uric acid, CRP, BNP, and troponin were found to be the independent predictors of SR development (CHA_2_DS_2_-VASc, OR = 0.288, *p* < 0.001; SYNTAX, OR = 0.920, *p*=0.007; uric acid, OR = 0.868, *p*=0.005; CRP, OR = 0.939, *p*=0.001; BNP, OR = 0.998, *p*=0.004; troponin, OR = 0.991, *p*=0.001). However, although LVEF was found to be statistically significant in both groups, it was not included in the logistic regression model since decreased LVEF was considered to be associated with the outcome rather than to be a predictor of SR (Table 2).

**Figure 2:**
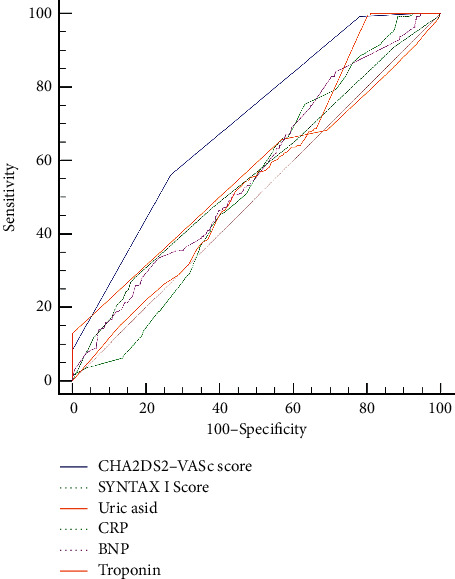
Receiver operating characteristic (ROC) curves of the predictors of spontaneous reperfusion.

The ROC curve analysis provided a cut-off value of ≤1 for the CHA_2_DS_2_-VASc score to predict the SR of STEMI patients with 56.1% sensitivity and 73.3% specificity (AUC: 0.703, 95% CI: 0.681–0.725, *p* < 0.001) (Table 3). The pairwise comparison of ROC curves revealed that the CHA_2_DS_2_-VASc score had statistically better discriminatory power than the remaining determinants for predicting SR in STEMI patients undergoing primary PCI (Table 4, Figure 2).

## 4. Discussion

The present study investigated the relationship between the CHA_2_DS_2_-VASc score and SR development in patients with STEMI who underwent CAG. The results indicated that the CHA_2_DS_2_-VASc and SYNTAX I scores were significantly lower in the SR group compared to the non-SR group, in-hospital mortality rates were significantly lower in the SR group, and the CHA_2_DS_2_-VASc score, uric acid, CRP, BNP, and troponin were found to be the independent predictors of SR.

Acute coronary syndromes remain a leading cause of morbidity and mortality worldwide [1]. STEMI is a clinical condition that arises from the rupture and erosion of the delicate atheromatous plaque followed by its complete obstruction because of thrombus formation. In patients presenting with STEMI, early clinical evaluation is of paramount importance for diagnosis and treatment. In such patients, CAG is commonly used for diagnosis and treatment purposes, and SR is diagnosed as the presence of TIMI grade 3 flow in IRA on the baseline CAG [20].

There is no consensus in the literature regarding the correct timing of the intervention to be performed in STEMI patients with SR who underwent delayed PCI or emergency intervention, with some reporting similar clinical outcomes independent of the timing of the intervention. More importantly, the authors noted that patients who presented with the clinical signs of reperfusion achieved favorable outcomes, despite the lack of immediate intervention [21].

The presence of SR on pre-PCI angiography is an independent marker of high procedural success and has been associated with a smaller infarction area and a better short- and long-term prognosis [6, 22–26]. Accordingly, it should be recognized that early prediction of SR in STEMI patients is highly important. The incidence of SR in STEMI patients has been reported to be between 7% and 27% in the literature [5], and it was found to be 14.6% (239 out of 1,641 patients) in our study.

The CHA_2_DS_2_-VASc score is a simple, easy-to-remember, valid, and practical scoring system used for predicting cardioembolic risk in patients with nonvalvular AF and for determining the indications for anticoagulant therapy. In recent studies, this scoring system is a predictor of ischemic stroke and adverse cardiovascular events, including death in cases of CAD without AF. Additionally, it has been associated with mortality in patients with stable angina and ACS [27, 28]. A previous study suggested that the CHA_2_DS_2_-VASc score could be used for predicting the severity of CAD [28]. Moreover, another study indicated that the CHA_2_DS_2_-VASc score could be a marker of the no-reflow phenomenon in both STEMI and NSTEMI patients [29, 30]. In some other studies, the CHA_2_DS_2_-VASc score has been reported to be a predictor of adverse cardiovascular events and mortality in patients with Takotsubo syndrome without AF [31]. Similarly, it has also been shown that the CHA_2_DS_2_-VASc score is a predictor of stroke and death in patients with sick sinus syndrome following pacemaker implantation [32]. Additionally, Yilmaz et al. showed that the CHA_2_DS_2_-VASc score was a predictor of in-stent restenosis [33].

To our knowledge, there has been no study in the existing literature investigating the relationship between SR development and the CHA_2_DS_2_-VASc score in STEMI patients. The present study evaluated a total of 1,641 consecutive patients who underwent CAG because of STEMI and revealed that the CHA_2_DS_2_-VASc score was significantly lower in the SR group compared to the non-SR group. Moreover, multivariate analysis indicated that the CHA_2_DS_2_-VASc score, uric acid, CRP, BNP, and troponin were found to be the independent predictors of SR development.

The SYNTAX score is commonly used in the quantification of the severity of CAD [17]. This scoring system has been shown to be an independent predictor of major adverse cardiac and cerebral events (MACCE) in patients treated with coronary artery bypass grafting (CABG) and PCI [30, 34]. Moreover, increased SYNTAX scores have been reported to be associated with contrast nephropathy and new-onset AF [35, 36]. On the other hand, the severity of CAD determined by the SYNTAX score is associated with the arterial stiffness assessed by pulse wave velocity (PWV) [37]. A previous study also showed that the SYNTAX score and the Killip classes from 2 to 4 were significantly higher in STEMI patients without SR [38]. In the present study, the SYNTAX score was significantly lower in the SR group compared to the non-SR group.

A study showed that in-hospital mortality was almost nonexistent in patients with SR, while it was remarkably high in patients without SR [6, 21–25]. Similarly, in our study, in-hospital mortality rate was significantly lower in the SR group compared to the non-SR group (0% *vs.* 6.7%, *p* < 0.001).

Terkelsen et al. reported that patients with spontaneous ST-resolution had a lower peak level of troponin and a better LVEF value [26]. Additionally, it is commonly known that the detection of spontaneous ST-resolution on an electrocardiogram indicates microvascular circulation, and thus, it is more desirable than the detection of SR on angiography [39]. In our study, the LVEF value was significantly higher in the SR group compared to the non-SR group, whereas troponin level was lower in the SR group compared to the non-SR group.

### 4.1. Limitations of the Study

Retrospective nature is the major limitation of the present study. Larger-scale prospective studies with more patients should be done to maintain more accurate prognostic information.

## 5. Conclusion

Our results indicated that lower CHA_2_DS_2_-VASc and SYNTAX scores are associated with SR development in STEMI patients. To the best of our knowledge, this is the first study to examine the relationship between the CHA_2_DS_2_-VASc score and SR development in STEMI patients. The ROC analysis indicated that the CHA_2_DS_2_-VASc score at a cut-off value of ≤1 may predict SR development. Based on the finding, we conclude that the CHA_2_DS_2_-VASc score is a useful predictor of SR development in STEMI patients. Furthermore, larger-scale studies are needed to pave the way for widespread use of this scoring system in clinical practice.

## Figures and Tables

**Table 1:** Demographic and clinical characteristics.

Variables	SR (+) (*n* = 239)	SR (−) (*n* = 1402)	*p*
Age (years)	62.59 ± 12.62	60.53 ± 12.30	**0.017**
Male gender (*n*, (%))	173 (72)	987 (70)	0.533
BMI (kg/m^2^)	25.55 ± 3.42	24.65 ± 2.99	0.487
HT (*n* (%))	157 (65)	981 (69)	0.185
DM (*n* (%))	51 (21)	391 (27)	**0.035**
HLD (*n* (%))	110 (46)	574 (41)	0.565
Smokers (*n* (%))	138 (58)	785 (56)	0.777
LVEF (%)	41.01 ± 7.51	36.01 ± 6.63	**0.024**
Emergency treatment			
ASA + clopidogrel (*n* (%))	144 (60)	1003 (71)	**0.002**
ASA + ticagrelor (*n* (%))	60 (25)	239 (17)	
ASA + prasugrel (*n* (%))	35 (14)	160 (11)	
CHA_2_DS_2_-VASc score	1.36 ± 0.64	2.01 ± 0.80	**<0.001**
SYNTAX I score	15.51 ± 5.94	17.08 ± 8.29	**<0.001**
In-hospital mortality (*n* (%))	1 (0.4)	81 (5.8)	**<0.001**
Laboratory parameters			
Total cholesterol (mg/dl)	185.45 ± 49.41	188.73 ± 48.62	0.406
LDL (mg/dl)	124.50 ± 43.56	126.41 ± 42.50	0.580
HDL (mg/dl)	38.21 ± 12.60	38.33 ± 12.62	0.908
Triglyceride (mg/dl)	131.69 ± 61.98	127.43 ± 57.25	0.367
Uric acid (mg/dl)	4.59 ± 2.05	4.92 ± 1.54	**0.019**
Creatinine (mg/dl)	0.93 ± 0.35	0.89 ± 0.30	**0.050**
Hemoglobin (g/dl)	13.65 ± 2.01	13.48 ± 1.98	0.234
Hematocrit (%)	41.55 ± 5.73	41.13 ± 5.10	0.266
Platelet count (10^3/uL)	268.56 ± 104.03	260.24 ± 76.65	0.164
WBC (10^3/uL)	12.00 ± 3.02	12.87 ± 5.08	**<0.001**
TSH (uIU/ml)	1.58 ± 0.75	1.58 ± 0.72	0.995
CRP (mg/L)	9.02 ± 4.34	9.95 ± 3.73	**0.002**
BNP (pg/ml)	153 (50–581)	243 (60–720)	**0.001**
Troponin (ng/ml)	13 (2–50)	21 (2–50)	**0.008**

BMI: body mass index, HT: hypertension, DM: diabetes mellitus, HLD: hyperlipidemia, LVEF: left ventricular ejection fraction, ASA: acetylsalicylic acid, SYNTAX: Synergy Between Percutaneous Coronary Intervention with Taxus and Cardiac Surgery, LDL: low-density lipoprotein, HDL: high-density lipoprotein, WBC: white blood cell count, TSH: thyroid stimulating hormone, CRP: C-reactive protein, and BNP: brain natriuretic peptide.

**Table 2:** Univariate and multivariate analyses of predictors of spontaneous reperfusion on logistic regression analysis.

Variable	Univariate			Multivariate		
	OR	95%CI	*p*	OR	95%CI	*p*
Age	1.013	1.002–1.025	**0.017**	1.020	0.950–1.090	0.208
Gender	1.102	0.812–1.496	0.533			
BMI	0.988	0.765–1.195	0.618			
HT	0.822	0.615–1.099	0.185			
DM	0.701	0.504–0.976	**0.036**	0.850	0.701–1.015	0.321
Smoke	0.845	0.573–1.249	0.402			
Treatment^+^			**0.002**			0.063
ASA + Ticagrelor	1.524	1.016–2.285	**0.042**	1.506	0.959–2.364	0.075
ASA + Prasugrel	1.749	1.254–2.439	**0.001**	1.434	0.991–2.074	0.056
CHA_2_DS_2_-VASc score	0.284	0.226–0.357	**<0.001**	0.288	0.227–0.364	**<0.001**
SYNTAX I score	0.974	0.957–0.992	**0.005**	0.920	0.903–0.937	**0.007**
Uric acid	0.867	0.787–0.955	**0.004**	0.868	0.785–0.959	**0.005**
Creatinine	1.617	1.059–2.469	**0.026**	1.321	0.822–1.796	0.241
TSH	0.999	0.676–1.474	0.995			
CRP	0.942	0.910–0.974	**0.001**	0.939	0.904–0.976	**0.001**
BNP	0.998	0.997–0.999	**0.001**	0.998	0.997–0.999	**0.004**
Troponin	0.990	0.985–0.994	**<0.001**	0.991	0.986–0.997	**0.001**

BMI: body mass index, HT: hypertension, DM: diabetes mellitus, ASA: acetylsalicylic acid, SYNTAX: Synergy Between Percutaneous Coronary Intervention with Taxus and Cardiac Surgery, TSH: thyroid stimulating hormone, CRP: C-reactive protein, BNP: brain natriuretic peptide, −2Log likelihood: 117,889, Nagelkerke *R*^2^ = 0.357, ^*∗*^*p* value <0.05 was considered significant, CI: confidence interval, and OR: ddds ratio. ^+^ASA+Clopidogrel is reference category.

**Table 3:** Comparison of receiver operating characteristic (ROC) curves for spontaneous reperfusion.

Variable	Cut-off ^*∗*^	AUC	SE^a^	95% CI^b^	Sensitivity, %	Specificity, %	*p*
CHA_2_DS_2_-VASc score	≤1	0.703	0.0152	0.681–0.725	56.1	73.3	**<0.001**
SYNTAX I score	≤19.5	0.534	0.0178	0.510–0.558	75.3	36.8	**0.05**
Uric acid	≤2	0.562	0.0208	0.538–0.586	12.9	100	**0.003**
CRP	≤9	0.560	0.0205	0.536–0.584	27.6	83.5	**0.003**
BNP	≤678	0.568	0.0196	0.544–0.592	84.1	28.6	**0.0005**
Troponin	≤52	0.555	0.0181	0.530–0.579	99.6	19.9	**0.003**

^a^Standard error by DeLong et al, ^b^confidence interval with binomial exact, and ^*∗*^ cut-off values were determined by the Youden index.

**Table 4:** Pairwise comparison of CHA2DS2-VASc score with other determinants of spontaneous reperfusion.

Variable	Differences between areas	SE	95% CI	*Z* statistic	*p*
CHA_2_DS_2_-VASc score∼SYNTAX I score	0.169	0.024	0.121–0.217	6.902	**<0.001**
CHA_2_DS_2_-VASc score∼uric asid	0.141	0.025	0.0915–0.191	5.574	**<0.001**
CHA_2_DS_2_-VASc score∼CRP	0.143	0.025	0.0934–0.192	5.654	**<0.001**
CHA_2_DS_2_-VASc score∼BNP	0.135	0.025	0.0866–0.183	5.467	**<0.001**
CHA_2_DS_2_-VASc score∼troponin	0.149	0.024	0.103–0.196	6.258	**<0.001**

AUC: area under the curve, CI: confidence interval, SE: standard error, CRP: C-reactive protein, and BNP: brain natriuretic peptide.

## Data Availability

Data will be shared upon request.
